# The *Pseudomonas aeruginosa* PrrF sRNAs and PqsA promote biofilm formation at body temperature

**DOI:** 10.1128/jb.00507-25

**Published:** 2026-01-30

**Authors:** Rhishita Chourashi, Jacob M. Weiner, Tra-My Hoang, Khady Ouattara, Amanda G. Oglesby

**Affiliations:** 1 Department of Pharmaceutical Sciences, School of Pharmacy, University of Maryland 733678 , Baltimore, Maryland, USA; 2 Department of Microbiology and Immunology, School of Medicine, University of Maryland 12264, Baltimore, Maryland, USA; Dartmouth College Geisel School of Medicine, Hanover, New Hampshire, USA

**Keywords:** iron regulation, temperature regulation, PrrF, biofilm, PqsA, *Pseudomonas aeruginosa*

## Abstract

*Pseudomonas aeruginosa* is a gram-negative opportunistic pathogen that causes both acute and chronic infections in vulnerable populations. Treatment of *P. aeruginosa* infections is increasingly challenging due to multi-drug resistance, and biofilm formation during infection further increases antibiotic tolerance. Iron, which is sequestered by the host innate immune system, is also a key nutrient that is required for *P. aeruginosa* biofilm formation. The iron-responsive PrrF small regulatory RNAs (sRNAs) are key to *P. aeruginosa’s* iron starvation response, promote the production of the *Pseudomonas* quinolone signal (PQS) quorum sensing molecule, and are required for virulence in murine lung infection. Prior work showed that the PrrF sRNAs are dispensable for biofilm formation; however, these studies were performed using flow-cell biofilms grown at room temperature. Here, we demonstrate a temperature dependency for PrrF in *P. aeruginosa* biofilm formation: the genes for these sRNAs are required for optimal biofilm formation at 37°C but not 25°C. We further show that a ∆*pqsA* mutant, which lacks production of PQS and related metabolites, phenocopies the ∆*prrF* mutant. These studies demonstrate the importance of the PrrF sRNAs in *P. aeruginosa* biofilm formation at body temperature and reveal a previously underappreciated role of temperature in iron homeostasis and *P. aeruginosa* biofilm physiology.

**IMPORTANCE:**

Biofilm formation is a critical virulence trait for many microbial pathogens that confers tolerance to the host immune system and antimicrobials. *Pseudomonas aeruginosa* is an opportunistic pathogen that forms biofilms resulting in treatment failure. Iron is a known requirement for *P. aeruginosa* biofilm formation, yet the precise role of iron in biofilm physiology remains unclear. Here, we show that temperature alters the requirement for the PrrF small regulatory RNAs, key components of *P. aeruginosa’s* iron starvation response, for biofilm formation. Specifically, PrrF is required for the optimal formation of flow-cell biofilms at 37°C but not at 25°C, yet most flow-cell biofilm studies are conducted at 25°C. These results demonstrate a previously underappreciated role of temperature in *P. aeruginosa* biofilm physiology.

## INTRODUCTION

*Pseudomonas aeruginosa* is a versatile opportunistic pathogen that causes acute lung and blood infections in cancer patients and 10% of all hospital-acquired infections (1–4). *P. aeruginosa* also causes life-long chronic lung infections in individuals with cystic fibrosis (CF) and is a significant contributor to chronic wound infections in diabetics and surgical patients (5–7). *P. aeruginosa* is innately resistant to many therapeutic agents, and the emergence of multi-drug resistant strains of *P. aeruginosa* leads to persistent infections, longer hospital stays, and increased mortality rates (8). Biofilm formation during chronic infections further complicates treatment due to increased tolerance of these communities against antimicrobials (9). Biofilms contribute to many types of *P. aeruginosa* infections but are most problematic in chronic infections, such as those in the lungs of CF patients. Defining the regulatory pathways that contribute to *P. aeruginosa* biofilm formation may aid in the identification of novel anti-pseudomonal therapeutics that can improve treatment outcomes for chronic *P. aeruginosa* infections.

Iron is a critical determinant of *P. aeruginosa* virulence and biofilm formation (10–12), and disrupting iron homeostasis may yield novel targets for drug development. Iron is sequestered by host proteins as part of the innate immune defense, and *P. aeruginosa* overcomes iron limitation during infection through the expression of multiple high-affinity iron uptake systems (13, 14). This includes the production of siderophores that scavenge insoluble ferric iron [Fe(III)] in aerobic environments, the uptake of ferrous iron [Fe(II)] in anaerobic environments, and the uptake and degradation of heme, a significant source of iron in the human host (15). Multiple studies have shown that disruption of either siderophore or heme uptake systems strongly attenuates *P. aeruginosa* virulence (10, 12, 16, 17). However, the importance of each system appears to vary among different infection models (reviewed by Cornelis and Dingemans [18]), with Fe(II) and heme uptake more predominant in chronic infections (19, 20). This is likely due to reduced oxygen availability in biofilm communities, which are characteristic of chronic infections, resulting in greater ratios of Fe(II) to Fe(III) (21) and reducing the need for siderophore synthesis. To date, studies examining the role of specific iron uptake systems in *P. aeruginosa* biofilms are largely limited to the impact of siderophores (22). Specifically, the high-affinity siderophore pyoverdine, which is conserved across all the pseudomonads, is required for *P. aeruginosa* biofilm formation. In contrast, pyochelin, a lower affinity siderophore produced during modest iron starvation, is not required.

*P. aeruginosa* budgets the use of iron during infection by down-regulating the expression of Fe-dependent metabolic pathways. This function is largely mediated by the PrrF small regulatory RNAs (sRNAs), which are required for virulence in an acute murine lung infection model (23, 24). The PrrF sRNAs are produced in low iron conditions and negatively affect the levels of numerous mRNAs coding for non-essential, iron-containing metabolic proteins (25, 26). In doing so, PrrF spares the use of iron for only the most critical processes when this nutrient becomes limiting. The PrrF sRNAs are expressed by clinical isolates from acute and chronic infections (24, 26), and high levels of the PrrF sRNAs are detected in sputum isolated from CF patients, verifying they are produced during chronic CF lung infections (19). While much has been learned about the broad impact of PrrF on cell physiology and virulence, how the PrrF sRNAs contribute to survival and biofilm formation during chronic infections remains unclear.

Prior studies showed that the PrrF sRNAs promote production of the *Pseudomonas* quinolone signal (PQS) quorum sensing molecule by repressing expression of iron-containing proteins that degrade the PQS precursor, anthranilate (26). This regulation occurs through a direct interaction of the PrrF sRNAs with the *antR* mRNA, encoding a LysR-type activator of the genes encoding anthranilate dioxygenase (*antABC*) (27). As a result, PrrF blocks degradation of anthranilate, thus sparing this metabolite for PQS production (26). The PQS biosynthetic pathway is initiated by PqsA and mediates the synthesis of at least 55 distinct alkyl-quinolone (AQ) metabolites (28); all AQs that we have tested to date are dependent on the PrrF sRNAs for their full production (27). AQs can largely be divided into three groups based on distinct structures and biological activities of their quinolone moiety. PQS and HHQ function as quorum sensing molecules that bind to the cytosolic PqsR (*aka* MvfR) response regulator to co-induce virulence and biofilm gene expression (29). PQS also functions as an iron chelator, though the biological significance of this activity remains unclear (30, 31). HQNO is an inhibitor of cytochrome *bc_1_* activity and thus causes autolysis in *P. aeruginosa* cultures, leading to the release of extracellular DNA that contributes to the biofilm matrix (28, 32, 33). Thus, the ability of PrrF to promote AQ production may contribute to *P. aeruginosa* biofilm formation.

Despite the accepted role of iron in *P. aeruginosa* biofilm physiology, it remains largely unknown how the PrrF sRNAs function within *P. aeruginosa* biofilms. In this vein, the work described herein challenges a conclusion that has been held in the *P. aeruginosa* biofilm community for 20 years: that the PrrF sRNAs are not required for biofilm formation (22). Here, we show that the PrrF sRNAs are indeed required for biofilm formation in a flow-cell system but that there is a temperature dependency for this phenotype. Specifically, the ∆*prrF* mutant is defective for flow cell biofilm formation at 37°C (body temperature) but not at 25°C (environmental temperature). We further show that the ∆*pqsA* mutant phenocopies the ∆*prrF* mutant, supporting a model wherein PrrF-mediated AQ production contributes to biofilm formation. Altogether, these findings indicate that regulatory pathways affecting *P. aeruginosa* biofilm formation at environmental and body temperatures are distinct and demonstrate a key role for the PrrF sRNAs in biofilm formation at body temperature.

## RESULTS

### The *prrF* promoter is transcriptionally active during low iron 37°C biofilm growth

**Fig 1:**
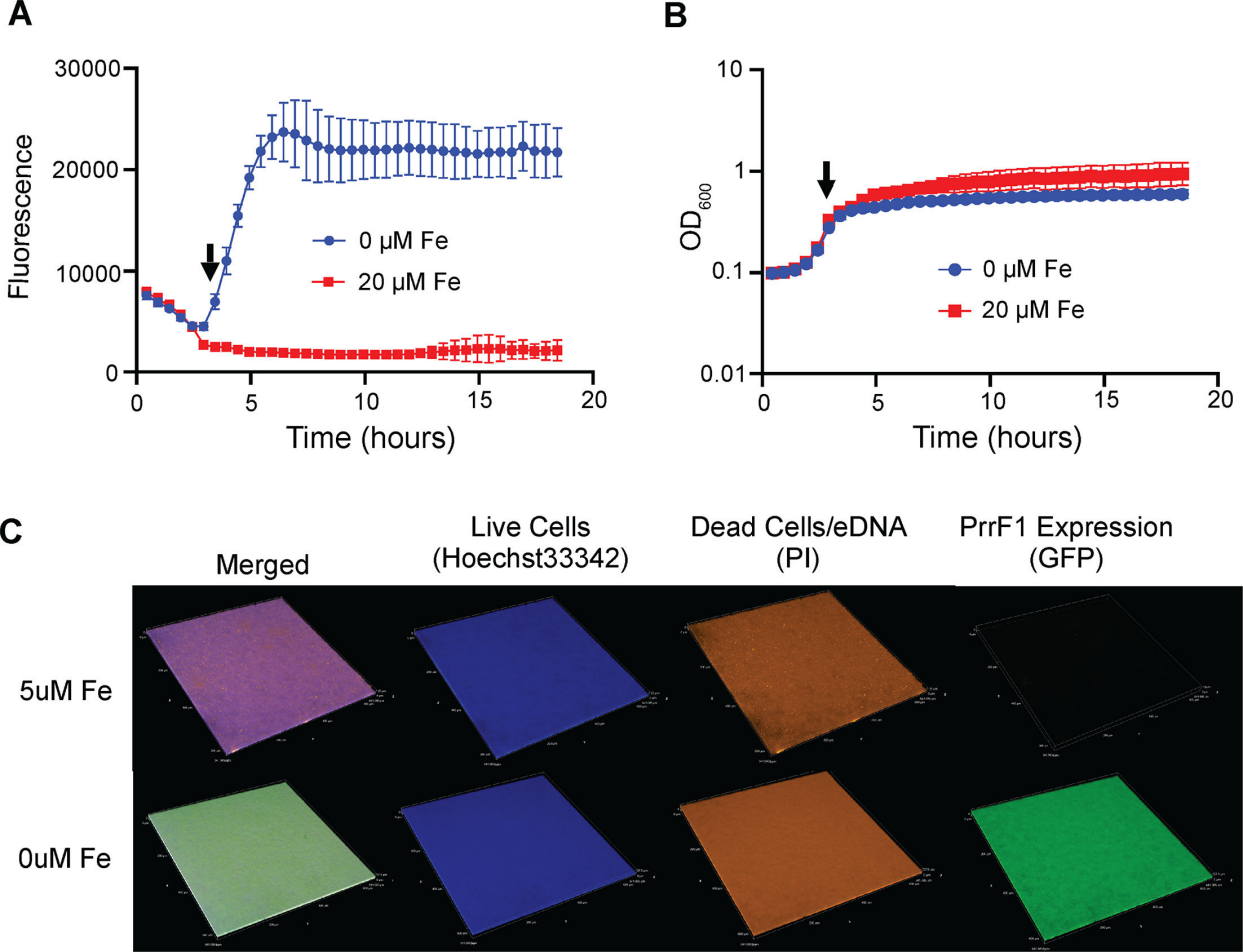
The P*_prrF1_* promoter is responsive to iron starvation in flow-cell biofilms. (**A, B**) Activity of the P*_prrF1_:gfp* reporter is repressed by supplementation of DTSB media with 20 µm FeSO_4_. Arrows on both graphs show the time (3.5 h) at which fluorescence starts to increase. (**A**) Fluorescence normalized to growth (OD_600_). (**B**) Growth curve of P*_prrF1_:gfp* reporter strain. (**C**) Representative confocal images showing iron repression and upregulation of PrrF1 promoter activity through GFP expression (green fluorescence) in flow-cell biofilms grown with or without 5 µm FeSO_4_, respectively. The Hoechst 33,342 (blue fluorescence) stains live biofilm cells; PI stains the eDNA (extracellular DNA) and dead cells. PI fluorescence is pseudo-colored orange. The three channels are merged in the left panels. Images are representative of at least five biological replicates shown in the supplementary materials (Fig. S1).

To begin studying iron homeostasis in *P. aeruginosa* biofilms, we generated a fluorescent transcriptional reporter using the *prrF1* promoter fused to green fluorescent protein (GFP). The reporter construct was introduced at the CTX *attB* site of strain PAO1 as previously described (34). We first confirmed that the resulting reporter strain was responsive to iron by growing it in high and low iron DTSB medium and tracking fluorescence in a BioTek Synergy HT plate reader at 37°C for 18 h (Fig. 1A). Fluorescence started to increase at 3.5 h into growth, corresponding with late logarithmic or early stationary phase (Fig. 1B). We next examined reporter activity in flow-cell biofilms formed after 48 h at 37°C. Confocal microscopy showed fluorescence throughout the area of the biofilm that was imaged and that supplementation of the flow cell media with 5 µM iron eliminated this signal (Fig. 1C; Fig. S1). We also confirmed that PAO1 biofilms grown under low iron conditions do not auto-fluoresce (Fig. S2). Together, these data indicate that the PrrF sRNAs are expressed and responsive to iron during biofilm formation at 37°C.

### The PrrF sRNAs are required for flow-cell biofilm formation at 37°C but not at 25°C

**Fig 2:**
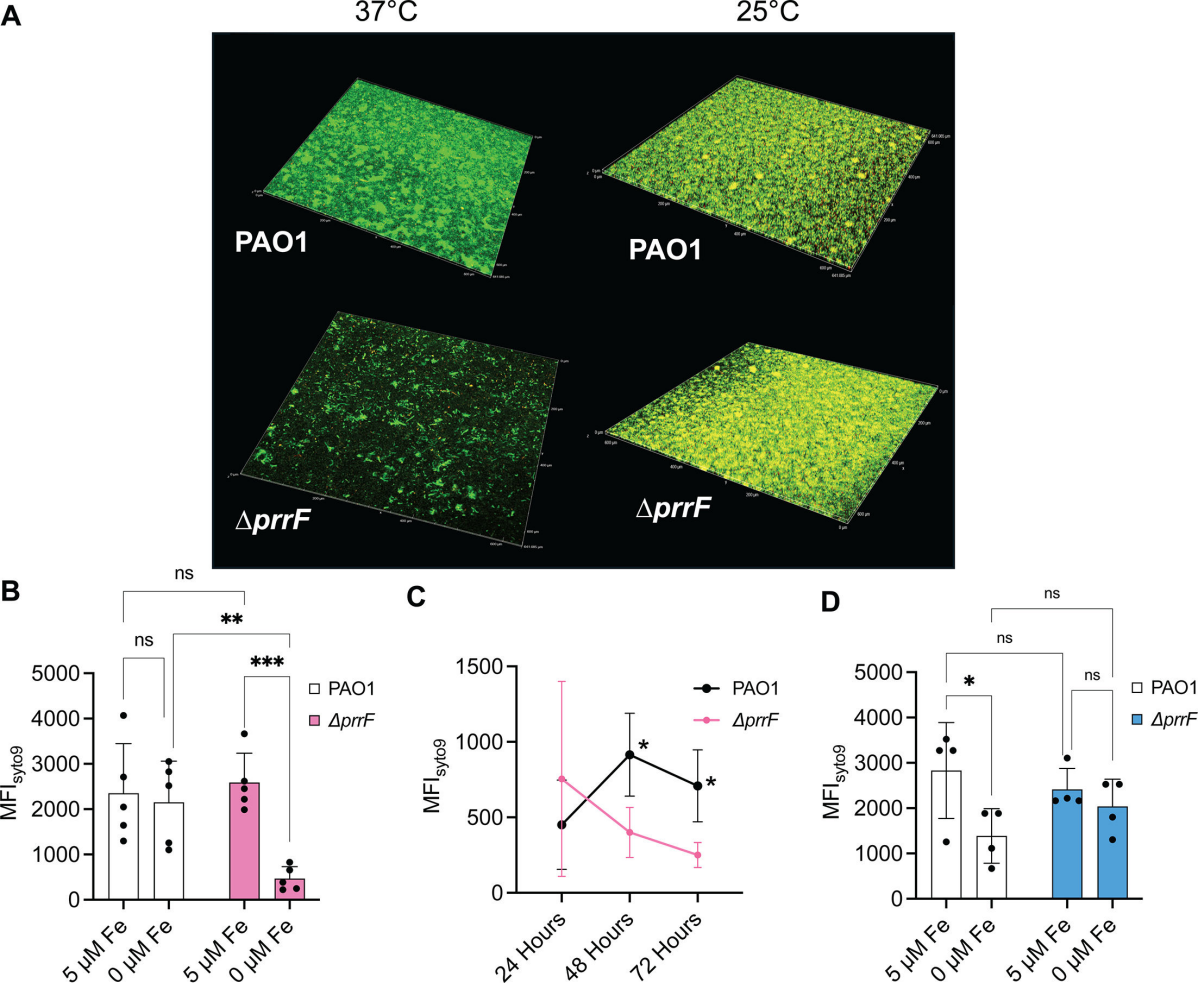
The ∆*prrF* mutant is defective for biofilm formation at 37°C but not 25°C. (**A**) Representative merged confocal microscopy images of the WT and ∆*prrF* strains grown in flow-cell biofilms at either 37°C (48 h) or 25°C (72 h) as described in the with or without supplementation of the medium with 5 µm FeSO_4_ (Materials and Methods). Live cells are stained with Syto9 (green) and eDNA and dead cells with PI (red). The red fluorescence of PI has been pseudo-colored with orange. (**B–D**) Quantitation of the MFI of the indicated conditions from at least five independent biofilm replicates grown at 37°C (**B–C**) or 25°C (**D**) for 48 h (**B**), 24–72 h (**C**), or 72 h (**D**). Results were represented as mean ± SD. Individual data points for all graphs are represented by a solid black circle. Asterisks indicate significant difference as indicated horizontal bars (* *P* < 0.05, ** *P* < 0.01, *** *P* < 0.001) as determined by two-way analysis of variance with Tukey’s *post hoc* test for multiple comparisons. ns, not statistically significant. Images from all biological replicates are shown in Supplementary Materials (Fig. S3 to S5).

**Fig 3:**
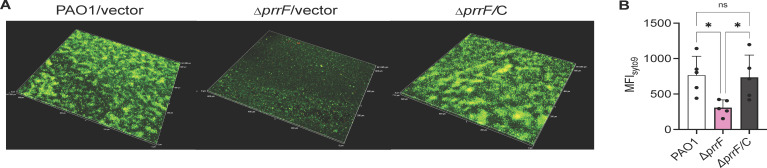
The defect in biofilm formation in ∆*prrF* mutant at 37°C is rescued by complementation. (**A**) Representative merged confocal microscopy images of the PAO1/vector (PAO1), ∆*prrF*/vector (∆*prrF*), and ∆*prrF*/C strains grown in flow-cell biofilms at 37°C without iron supplementation as described in Materials and Methods. Live cells are stained with Syto9 (green) and eDNA and dead cells with PI (red). (**B**) Quantitation of the MFI of the indicated conditions from six independent biofilm replicates. Results were represented as mean ± SD. Individual data points for all graphs are represented by a solid black circle. Asterisks indicate significant difference as indicated horizontal bars (* *P* < 0.05) as determined by one-way analysis of variance with Tukey’s *post hoc* test for multiple comparisons. ns, not statistically significant. Images from all biological replicates are shown in Supplementary Materials (Fig. S6).

Previous studies from Banin et al. demonstrated that a PAO1 ∆*prrF* mutant formed flow-cell biofilms similar to its wild-type parent (22). However, our above data indicate that the PrrF sRNAs are highly expressed in flow-cell biofilms grown at 37°C, and our earlier 37°C biofilm studies using minimum biofilm eradication concentration plates suggested the ∆*prrF* mutant may exhibit altered cyclic di-GMP (c-di-GMP) signaling in biofilms (23). Since the flow-cell biofilms from Banin et al. were grown at 25°C, we wondered if growth at 37°C may result in a different outcome. We therefore grew and imaged flow-cell biofilms of PAO1 and the isogenic ∆*prrF* mutant as described above. Under these conditions, the ∆*prrF* mutant demonstrated a clear defect in biofilm formation as compared to PAO1 when not supplemented with iron (Fig. 2A and B; Fig. S3). Moreover, iron supplementation rescued biofilm formation of the ∆*prrF* mutant (Fig. 2B; Fig. S3). Analysis of biofilm formation over time demonstrated that the ∆*prrF* mutant defect becomes apparent after 24 h of growth (Fig. 2C; Fig. S4), suggestive of an early dispersal phenotype. Complementation of the ∆*prrF* mutant in *trans*, compared to isogenic vector-control strains, also restored biofilm formation to the ∆*prrF* mutant (Fig. 3; Fig. S5). To determine if temperature is a likely reason for our findings differing from Banin et al., we conducted a second series of flow-cell experiments at room temperature, imaging the biofilms at 72 h. At this temperature, the ∆*prrF* mutant consistently formed biofilms that were either indistinguishable or slightly denser than that of the PAO1 parent strain (Fig. 2A and D; Fig. S6). Combined, these data show that the PrrF sRNAs are specifically required for flow-cell biofilm formation at body temperature.

### PqsA promotes low iron biofilm growth

**Fig 4:**
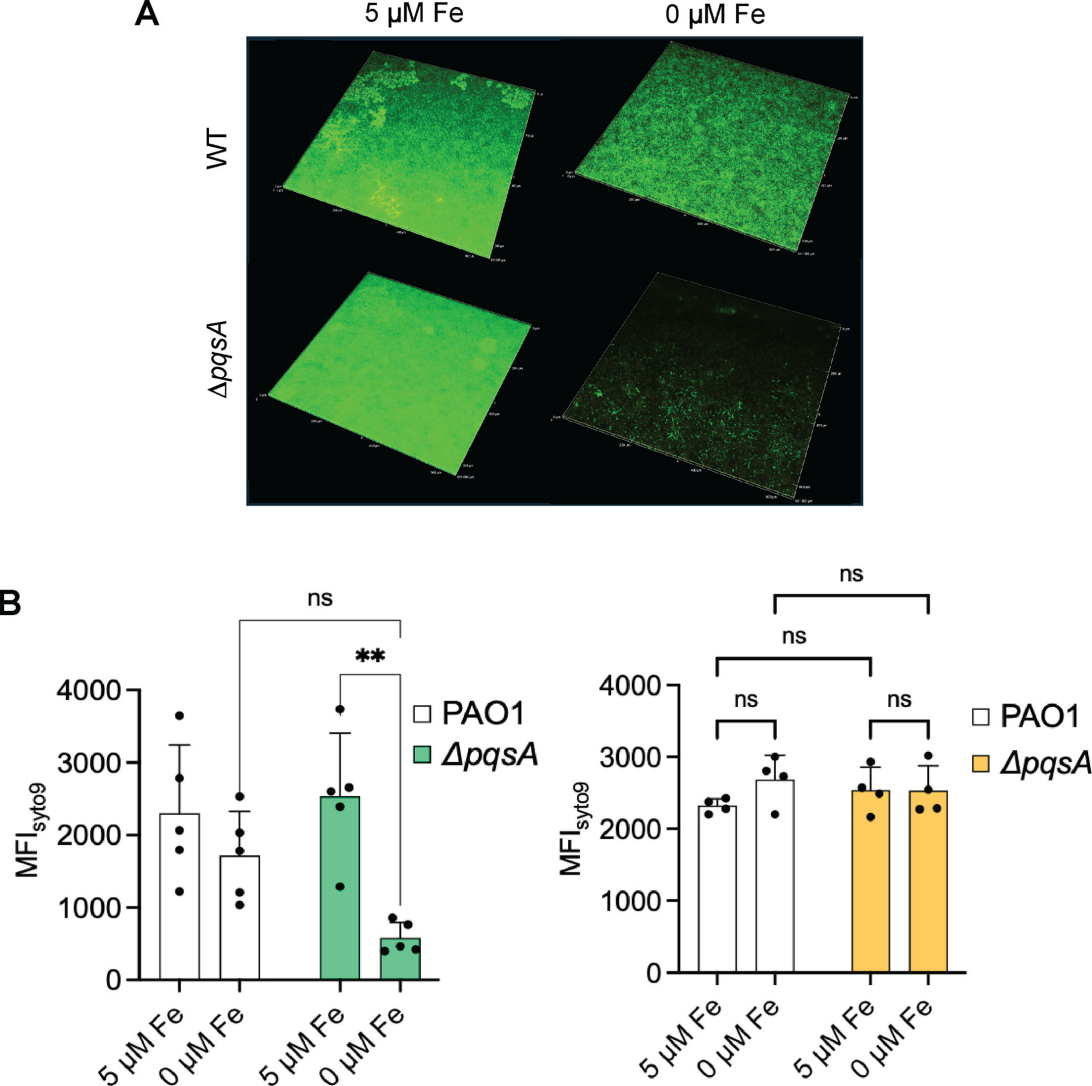
The ∆*pqsA* mutant exhibits a body temperature-dependent biofilm defect in low iron conditions. (**A**) Representative merged confocal microscopy images of the PAO1 and ∆*pqsA* strains grown in flow-cell biofilms at 37°C with or without supplementation of the medium with 5 µm FeSO_4_ as described in Materials and Methods. Live cells are stained with Syto9 (green) and eDNA and dead cells with PI (red). The red fluorescence of PI has been pseudo-colored with orange. (**B**) Quantitation of the MFI of the indicated strains from five independent biofilm replicates at either 37°C or 25°C. Results are represented as mean ± SD. Individual data points for all graphs are represented by a solid black circle. Asterisks indicate significant difference between indicated horizontal bars (** *P* < 0.01) as determined by two-way analysis of variance with Tukey’s *post hoc* test for multiple comparisons. ns, not statistically significant. Images from all biological replicates are shown in Supplementary Materials (Fig. S7 and S8).

We next examined the impact of PQS and related AQ metabolites, which require PrrF for their full production, on biofilm growth in low iron conditions. For this, we employed a *pqsA* deletion that lacks production of all AQ metabolites (28, 35). The ∆*pqsA* mutant grown in low iron conditions showed a similar defect in biofilm formation to the ∆*prrF* mutant, and iron similarly restored biofilm formation to the ∆*pqsA* mutant (Fig. 4; Fig. S7). Also similar to the ∆*prrF* mutant, the ∆*pqsA* mutant showed no defect in biofilm formation when grown at 25°C (Fig. 4B; Fig. S8). These data are consistent with a model wherein the ∆*prrF* mutant defect in biofilm formation is due to the lack of AQ production.

### PrrF negatively affects known PrrF-regulated transcripts at 25°C

**Fig 5:**
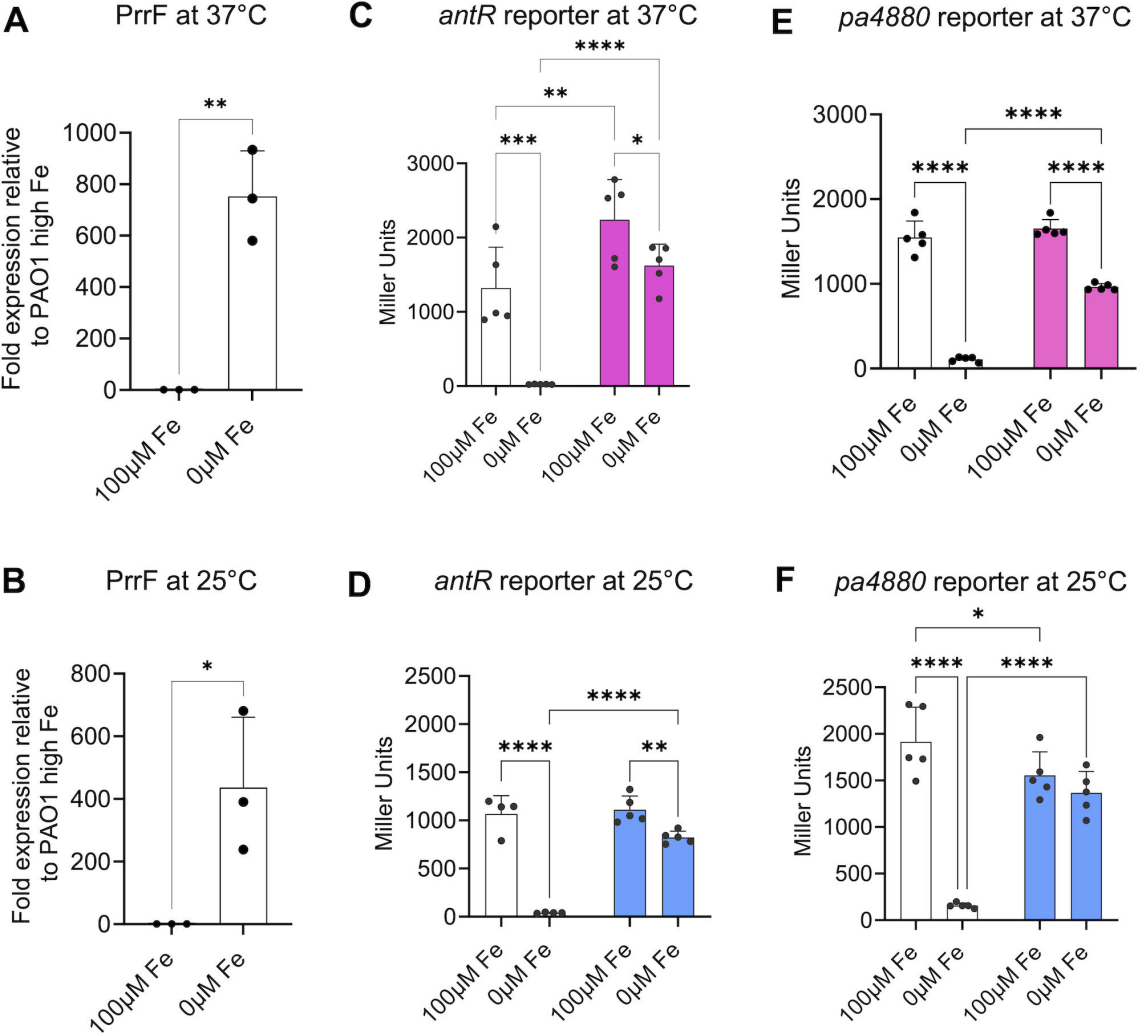
PrrF expression and regulation of two known targets is maintained at 25°C. The indicated strains were inoculated into DTSB, with or without supplementation of 100 μM of FeCl_3_, and incubated for 18 h at either 25°C or 37°C. Cultures were incubated under shaking conditions at 250 rpm. White bars indicate wild type; colored bars (pink and blue in **C, E** or **D, F**, respectively) indicate the ∆*prrF* mutant. (**A, B**) RNA was isolated and used for real-time PCR as indicated in Materials and Methods. qPCR results represent means ± SD of three biological replicates, with three technical replicates included for each biological replicate. (**C–F**) Reporter assays were performed by using the indicated strains containing the *PantR_antR+UTR_-LacZ^-SD^* and *Ppa4880_pa4880+UTR_-LacZ^-SD^* reporter fusions. Cultures were subjected to β-galactosidase activity assay as described in Materials and Methods. Reporter assay data represents mean ± SD of five biological replicates. Individual data points for all graphs are represented by a solid black circle. Asterisks indicate significant difference between indicated horizontal bars (* *P* < 0.05, ** *P* <0.005, *** *P* < 0.001, **** *P* < 0.0001) as determined by unpaired *t* test for the qPCR data and two-way analysis of variance with Fisher’s least significant difference (LSD) test for multiple comparisons for the reporter assays.

We next examined whether decreased temperature affected PrrF expression and/or function in planktonic cultures. Real-time quantitative PCR (qPCR) demonstrated that the PrrF sRNAs are expressed in low iron conditions at 25°C, with PrrF C_T_ levels that are similar to what we observe when PAO1 is grown in low iron media at 37°C (Fig. 5A and B). Similar to earlier reports at 37°C, we also observed robust repression of the PrrF sRNAs when PAO1 was grown in medium supplemented with 100 µM FeCl_3_ at 25°C (19, 23, 24, 36, 37). Reporters for two distinct PrrF target mRNAs*—pa4880* and *antR* (25)—were strongly de-repressed in the ∆*prrF* mutant in iron-depleted media at both 37°C and 25°C (Fig. 5C through F), demonstrating PrrF still represses expression of these genes at 25°C. While these data do not rule out PrrF regulates the same gene targets in 25°C and 37°C during biofilm formation, these data do demonstrate that the PrrF sRNAs are functional and regulate at least some of the same targets at environmental temperature.

## DISCUSSION

This study demonstrates the importance of the PrrF sRNAs and PqsA in *P. aeruginosa* biofilm formation. While the specific mechanisms underlying these findings remain unknown, this is a critical discovery considering that many flow-cell biofilm studies with *P. aeruginosa*, particularly those regarding iron, have been conducted at 25°C (22). In the current study, we demonstrate that PrrF affects iron-dependent biofilm formation at 37°C but not 25°C. We further show that the iron regulation of biofilm formation at 37°C occurs in a PqsA-dependent manner, consistent with a model in which the ∆*prrF* defect in biofilm formation is due to a loss of AQ production. While we have not definitively confirmed that the PrrF sRNAs are expressed in biofilms formed at 25°C, we do provide evidence that these sRNAs are expressed at room temperature and are functional in planktonic room temperature cultures. Biofilm formation is dependent upon the production of the second messenger c-di-GMP (38), which has been linked to iron regulation in *P. aeruginosa* (39). Our own work shows that induction of *P. aeruginosa* biofilm formation by tobramycin in static conditions, a phenomenon that is dependent on c-di-GMP signaling (40), is also dependent on the PrrF sRNAs (24). This finding was also observed in a ∆*prrF* mutant constructed in a PAO1 derivative strain, H103, as well as an isogenic ∆*pqsA* mutant (41). Collectively, these studies indicate that *P. aeruginosa* community behaviors are mediated by PrrF-dependent AQ production, presumably by modulating c-di-GMP signaling pathways.

It is notable that the dependence of both PrrF and PqsA for flow cell biofilm formation is not observed at 25°C. *P. aeruginosa* is one of the few well-studied pseudomonads that can grow at body temperature and cause infection, and it is the only *Pseudomonas* species that possesses the genes for AQ production. Temperature is a key factor for virulence gene regulation by many pathogens, yet few studies have addressed how temperature affects global gene expression in *P. aeruginosa* (42, 43). Two independent studies showed that *P. aeruginosa* significantly changes in its transcriptome when grown at 37°C versus 25°C (42, 43). One of these studies discovered a shift from pyoverdine to pyochelin siderophore gene expression at 25°C compared to 37°C, suggesting iron homeostasis plays a key role in the transition from environmental to body temperature (42). Additional studies have revealed that temperature alters *P. aeruginosa* phage gene expression as well as the matrix composition in *P. aeruginosa* biofilms (44, 45). These prior studies, combined with our current work, demonstrate the importance of considering temperature in *P. aeruginosa* biofilm physiology. Of key interest now is whether temperature impacts the production of AQs.

RNA thermometers have become increasingly appreciated in the role of temperature-dependent gene expression changes. These elements are often found in the 5′ untranslated regions (UTRs) of bacterial virulence genes and melt upon increased temperature, thereby altering access of RNA polymerase to the translational start site (46–48). To our knowledge, three RNA thermometers have been identified in *P. aeruginosa* mRNAs: these encode the RhlI and LasI quorum sensing synthases and PtxS which activates transcription of exotoxin A (49, 50). Notably, the *toxA* gene encoding exotoxin A is also induced by iron starvation (51), providing yet another link between temperature and iron regulation. These and other mechanisms likely alter the ability of certain genes to be expressed at either environmental or body temperature, potentially affecting interactions between PrrF and its target mRNAs. The role of RNA structure in PrrF-mediated biofilm formation is therefore a high priority for future studies regarding temperature-dependent changes in *P. aeruginosa* biofilm physiology.

It is important to note that findings from room temperature biofilm studies remain clinically significant even if they are distinct from what is observed at body temperature. *P. aeruginosa* is commonly acquired in hospital settings and has been identified in environmental reservoirs such as sink faucets (52) where it likely grows in biofilms. Moreover, the temperature in wounds is lower than that of the lungs (~30°C–33°C), while fever in response to infection raises the internal body temperature (up to 40°C). *P. aeruginosa* must therefore adapt to changing temperatures as they colonize and infect their host. A recent study from Alan Hauser’s group further showed that *P. aeruginosa* can be shed by mice back into the environment, resulting in its spread to uninfected cage-mates (53). While this study was restricted to mice, several studies suggest that *P. aeruginosa* can colonize the human gallbladder and intestines, potentially serving as a reservoir in clinical settings (54–59). Thus, the idea that clinical *P. aeruginosa* septicemia is a “dead-end” needs to be reconsidered, with temperature and biofilms playing potentially key roles in transmission to and from the host.

In closing, we have demonstrated that the iron-responsive PrrF sRNAs are important determinants of PAO1 biofilm formation at 37°C, and we predict that additional biofilm determinants of *P. aeruginosa* will vary between environmental and body temperatures. We anticipate that this work will provide an increased appreciation for the role of temperature in *P. aeruginosa* physiology and biofilm formation and the impact of temperature-dependent gene regulation in clinical settings.

## MATERIALS AND METHODS

### Bacterial strains and growth conditions

Strains are listed in Table S1 and were routinely grown overnight by streaking from freezer stocks in tryptic soy agar (Sigma, St Louis, MO) plates. The ∆*pilA* mutant was generously gifted by Prof. Christopher Pritchett, East Tennessee State University (*manuscript in revision*). For experiments, overnight cultures were inoculated with three to five isolated colonies from agar plates in 2 mL of Luria Bertani broth (LB, Sigma, St Louis, MO, USA). Brain heart infusion agar was for growth of *Pseudomonas* strains during genetic manipulations. Chemically defined medium (CDM) was prepared as previously described and supplemented with 1 mM CaCl_2_, 0.1 µM CuCl_2_, 0.1 µM NiCl_2_, 6 µM ZnCl_2_, and 0.3 µM MnCl_2_ (37). CDM was supplemented with 100 µM FeCl_3_ as indicated. *Escherichia coli* strains for cloning were grown in LB. Antibiotics were added in the following concentration: ampicillin, 100 μg/mL (*E. coli*); tetracycline, 10 or 15 μg/mL (*E. coli*); gentamicin 20 μg/mL (*E. coli*); irgasan 25 μg/mL, carbenicillin, 250 μg/mL (*P. aeruginosa*); tetracycline, 150 μg/mL (*P. aeruginosa*); and gentamicin 50 μg/mL (*P. aeruginosa*).

### Generation of reporter constructs

For the PrrF1 reporter, the promoter region of *prrF1* (P*_prrF1_*) was amplified by PCR using primers in Table S2 and the PAO1 genomic DNA as a template. The P*_prrF1_* insert was digested with restriction enzymes BamHI and HindIII and ligated into yeast vector pMQ37 (*aka* pLD2477; contains the coding region for GFP [60]) digested with the same restriction enzymes used for the *prrF1* promoter fragment. The ligated pMQ37-P*_prrF1_* plasmid was transformed into *E. coli* strain SM10, then purified, digested with BamHI and EcoRI, and ligated into the mini-CTX-P*_prrF1_-gfp* plasmid digested with the same enzymes. The resulting plasmid was transformed into *E. coli* strain SM10, then purified and transformed into *P. aeruginosa* PAO1 via electroporation. The pFLP plasmid (34) was transformed into PAO1 mini-CTX-P*_prrF1_-gfp* via electroporation to excise the integrated mini-CTX as previously described (61).

For the *pa4880* reporter, the promoter and 5′ UTR of *pa4880* were amplified by PCR using primers mentioned in Table S2 and the PAO1 genomic DNA as a template. The PCR product was cloned in a TA cloning vector PCR2.1 (Invitrogen), and the resulting plasmid was confirmed by sequencing. The promoter fragment was then digested by restriction enzymes EcoRI and HindIII, and the resulting fragment was ligated into the promoterless *lacZ* reporter plasmid mini-CTX-*lacZY*^-SD^ vector digested with the same enzymes to generate *P_pa4880+UTR_-lacZ^-SD^* plasmid. All reporter constructs were integrated into the *att* site for CTX phage on the PAO1 and ∆*prrF* chromosomes as previously described (61). Schematics of the reporter constructs are shown in Fig. S9.

### Real-time PCR

Real-time PCR (qPCR) was performed using three biological replicates of wild-type *P. aeruginosa* PAO1 and the isogenic deletion mutant ∆*prrF*. Strains were inoculated to an OD_600_ of 0.05 into CDM with or without 100 µM FeCl_3_ and grown for 18 h at 37°C in CDM supplemented with or without 100 µM FeCl_3_. RNA was isolated and DNase-treated, and cDNA was synthesized using reverse transcription kit (Promega) with 50 ng of total RNA as previously described (62). cDNA was analyzed by qPCR using Taqman (Takara). Primers and probes are listed in Table S1. The 16S rRNA was used as a normalizer. Quantification was carried out using standard curves generated from cDNA generated from 1:10 serial dilutions of RNA for each target.

### Reporter assays

Reporter constructs for *antR* and *pa4880* in PAO1 WT and *∆prrF* backgrounds were assayed for beta-galactosidase activity as previously described (27). Briefly, strains were inoculated to an OD_600_ of 0.05 into CDM with or without 100 µM FeCl_3_ and grown shaking for 18 h at either 25°C or 37°C. Cells were harvested by centrifugation, resuspended in potassium phosphate buffer (30 mM K_2_HPO_4_ and 20 mM KH_2_PO_4_), and the resulting cell suspension was diluted 1:10 into Z buffer (60 mM Na_2_HPO_4_, 40 mM NaH_2_PO_4_, 10 mM KCl, 1 mM MgSO_4_, and 50 mM β-mercaptoethanol). Chloroform and 1% SDS were also added into the reaction mixture. o-Nitrophenyl-β-D-galactopyranoside was then used as a substrate to begin the reaction. Once sufficient yellow color was observed, the reaction was stopped by addition of Na_2_CO_3_ to a final concentration of 0.25 M. The OD_420_ was measured for each sample, and the beta-galactosidase activity was calculated using Miller units formula [(1,000 × OD_420_) / (time × volume × OD_600_)].

### Biofilm growth

Strains were grown in LB overnight, then diluted to an optical density at 600 nm (OD_600_) of 0.05, and inoculated into a three-chamber flow cell (IBI Scientific, Dubuque, Iowa, USA). The chamber was placed upside down for 1 h to allow for bacterial adherence to the glass coverslip surface and then turned right side up, and a peristaltic pump (Ismatec, Wertheim, Germany) was used to pump 1% LB (vol/vol) supplemented with 1 mM CaCl_2_, 0.1 µM CuCl_2_, 0.1 µM NiCl_2_, 6 µM ZnCl_2_, and 0.3 µM MnCl_2_ through the flow cell at a rate of 3.5 mL/h at 25°C and 37°C for 72 and 48 h, respectively. Biofilms were stained with Hoechst 33,342 (live cells) or Syto9 (live cells) and propidium iodide (PI, extracellular DNA in the biofilm matrix) as indicated for 30 min, at which point flow with 1% LB was resumed to remove excess stain. The biofilms were imaged between the inlet and center of chambers as indicated using a Nikon A1 Confocal Microscope (Melville, NY, USA) and captured with NIS Elements software, using a 20× objective. The time course biofilm assay was performed with three flow cells that were incubated for either 24, 48, or 72 h at 37°C with a flow rate of 3.5 mL/h. Biofilms were then removed from the system and stained with Syto9 and PI for 30 min. Residual stain was washed out by reconnecting the flow cells to the system and allowing media to run at 3.5 mL/h for 1 h. Biofilms were imaged via confocal scanning laser microscopy using Nikon A1 Confocal Microscope and captured with NIS Elements software, using a 20× or 40× objective. Quantification of biofilms is achieved by measuring the fluorescence of the z stack showing the maximum fluorescence intensity (MFI) using Fiji ImageJ software.

## SUPPLEMENTAL MATERIAL

Fig. S1 jb.00507-25-s0001.pdf Confocal images showing iron-dependent regulation of the prrF1 promoter in flow-cell biofilms.

Fig. S2 jb.00507-25-s0002.pdf PAO1 biofilms do not auto-fluoresce.

Fig. S3 jb.00507-25-s0003.pdf Confocal images of all the biological replicates of biofilms of the indicated strains grown at 37°C.

Fig. S4 jb.00507-25-s0004.pdf Confocal images of all the biological replicates of time course biofilms of the indicated strains grown at 37°C.

Fig. S5 jb.00507-25-s0005.pdf Confocal images of biofilms of the indicated strains grown at 37°C.

Fig. S6 jb.00507-25-s0006.pdf Confocal images of biofilms of all the biological replicates of the indicated strains grown at 25°C.

Fig. S7 jb.00507-25-s0007.pdf Confocal images of biofilms of all the biological replicates of the indicated strains grown at 37°C.

Fig. S8 jb.00507-25-s0008.pdf Confocal images of biofilms of all the biological replicates of the indicated strains grown at 25°C.

Fig. S9 jb.00507-25-s0009.pdf Schematic of new reporter constructs.

Supplemental material jb.00507-25-s0010.pdf Legends for Figures S1 to S9, and Tables S1 and S2.

ASM does not own the copyrights to Supplemental Material that may be linked to, or accessed through, an article. The authors have granted ASM a non-exclusive, world-wide license to publish the Supplemental Material files. Please contact the corresponding author directly for reuse.
