# Supplementary material for: The Pseudomonas aeruginosa PrrF sRNAs and PqsA promote biofilm formation at body temperature
Source: J Bacteriol. 2026 Jan 30;208(2):e00507-25. doi: 10.1128/jb.00507-25 (PMC12918728; doi:10.1128/jb.00507-25)
Supplement: Fig. S1 — Confocal images showing iron-dependent regulation of the prrF1 promoter in flow-cell biofilms. [file jb.00507-25-s0001.pdf]

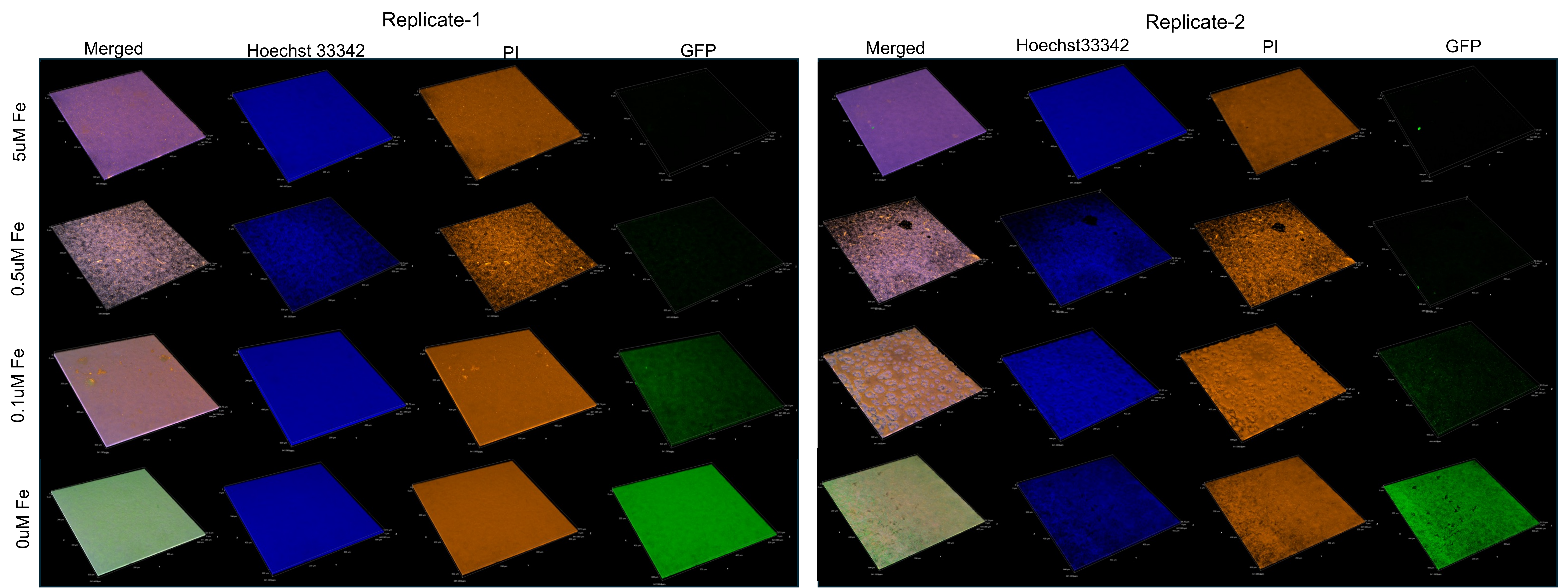

Replicate-1
Replicate-2
Merged
Hoechst 33342
PI
GFP
Merged
Hoechst33342
PI
GFP
5uM Fe
0.5uM Fe
0.1uM Fe
0uM Fe

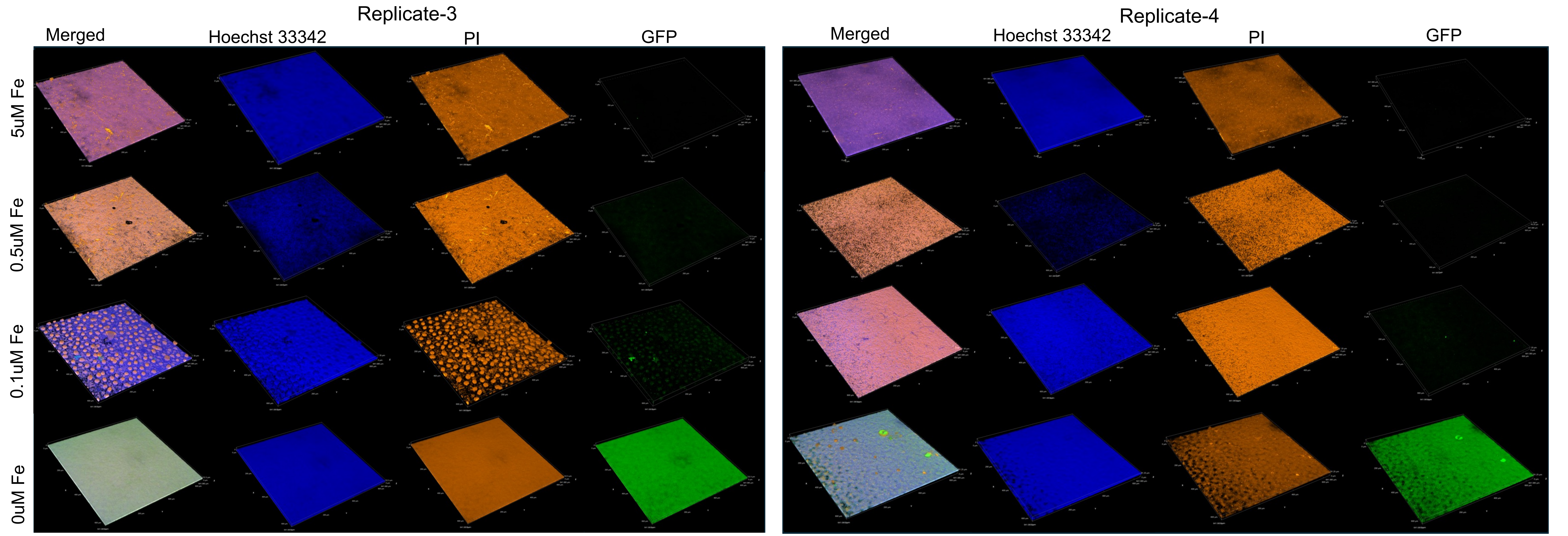

Replicate-3
Merged
Hoechst 33342
PI
GFP
Replicate-4
Merged
Hoechst 33342
PI
GFP
5uM Fe
0.5uM Fe
0.1uM Fe
0uM Fe

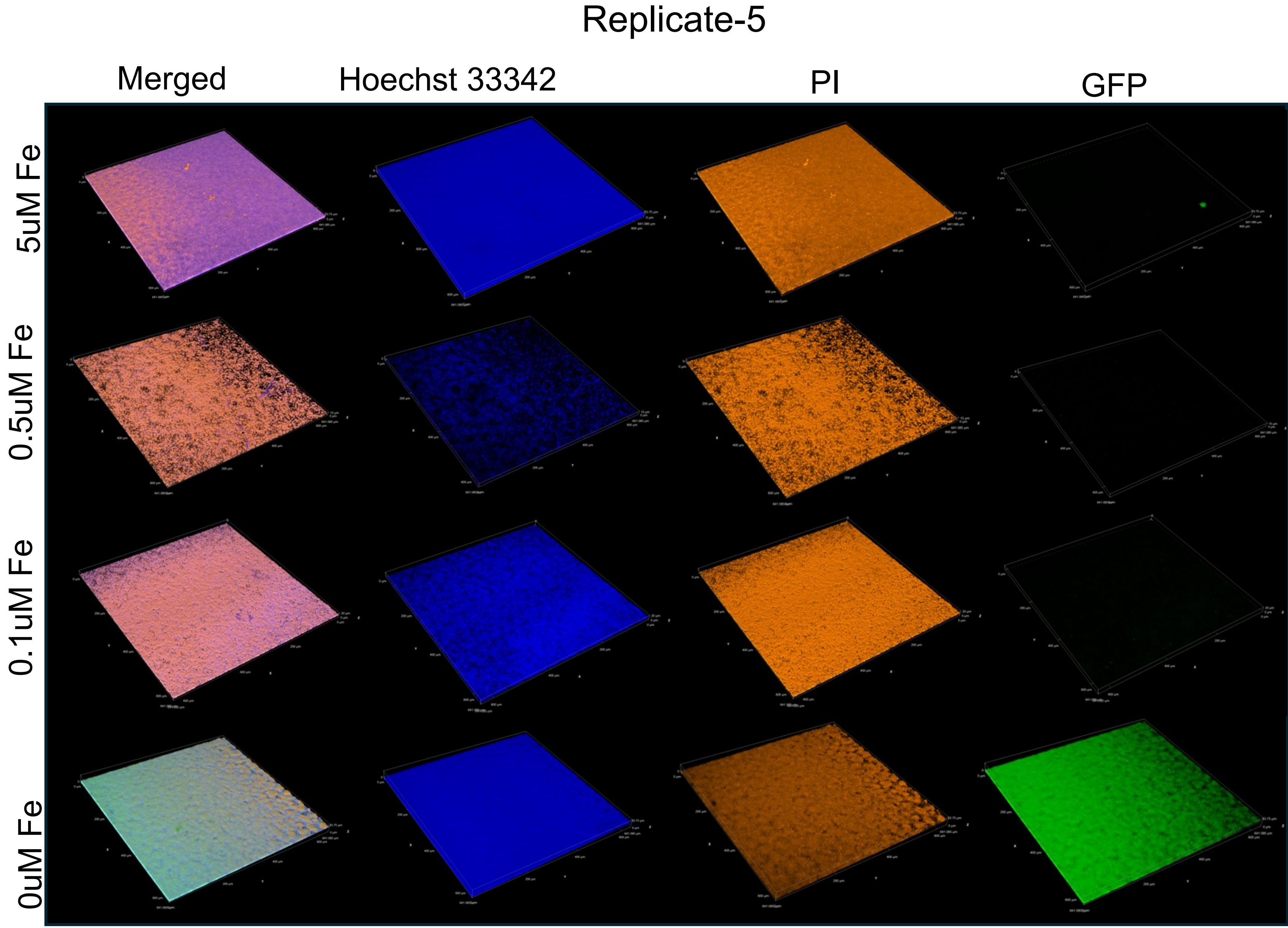
Replicate-5
Merged
Hoechst 33342
PI
GFP
5uM Fe
0.5uM Fe
0.1uM Fe
0uM Fe
