# Supplementary figures and images for: The Pseudomonas aeruginosa PrrF sRNAs and PqsA promote biofilm formation at body temperature
Source: J Bacteriol. 2026 Jan 30;208(2):e00507-25. doi: 10.1128/jb.00507-25 (PMC12918728; doi:10.1128/jb.00507-25)

Merged

Hoescht

PI

GFP

0uM Fe

R1

R2

R3

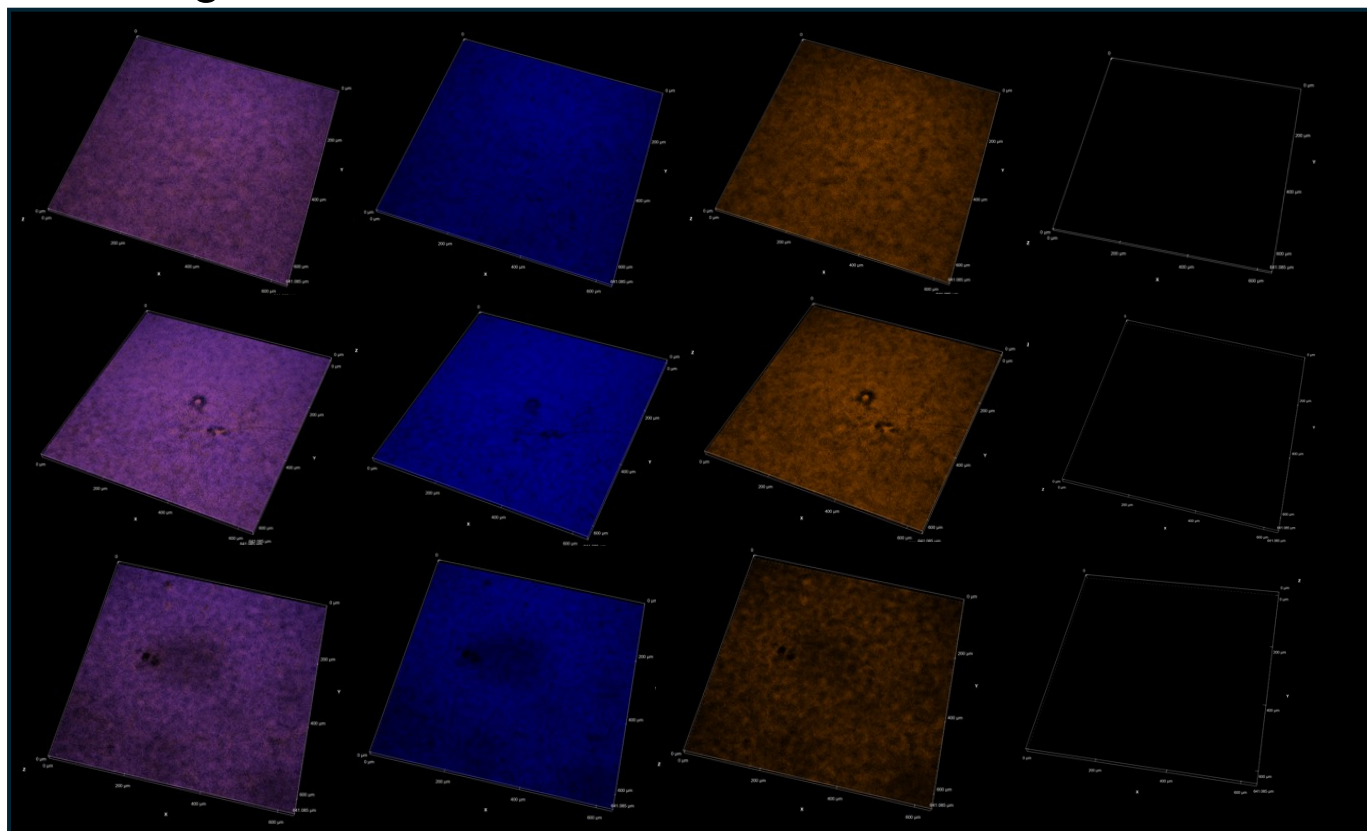

5uM Fe

R1

R2

R3

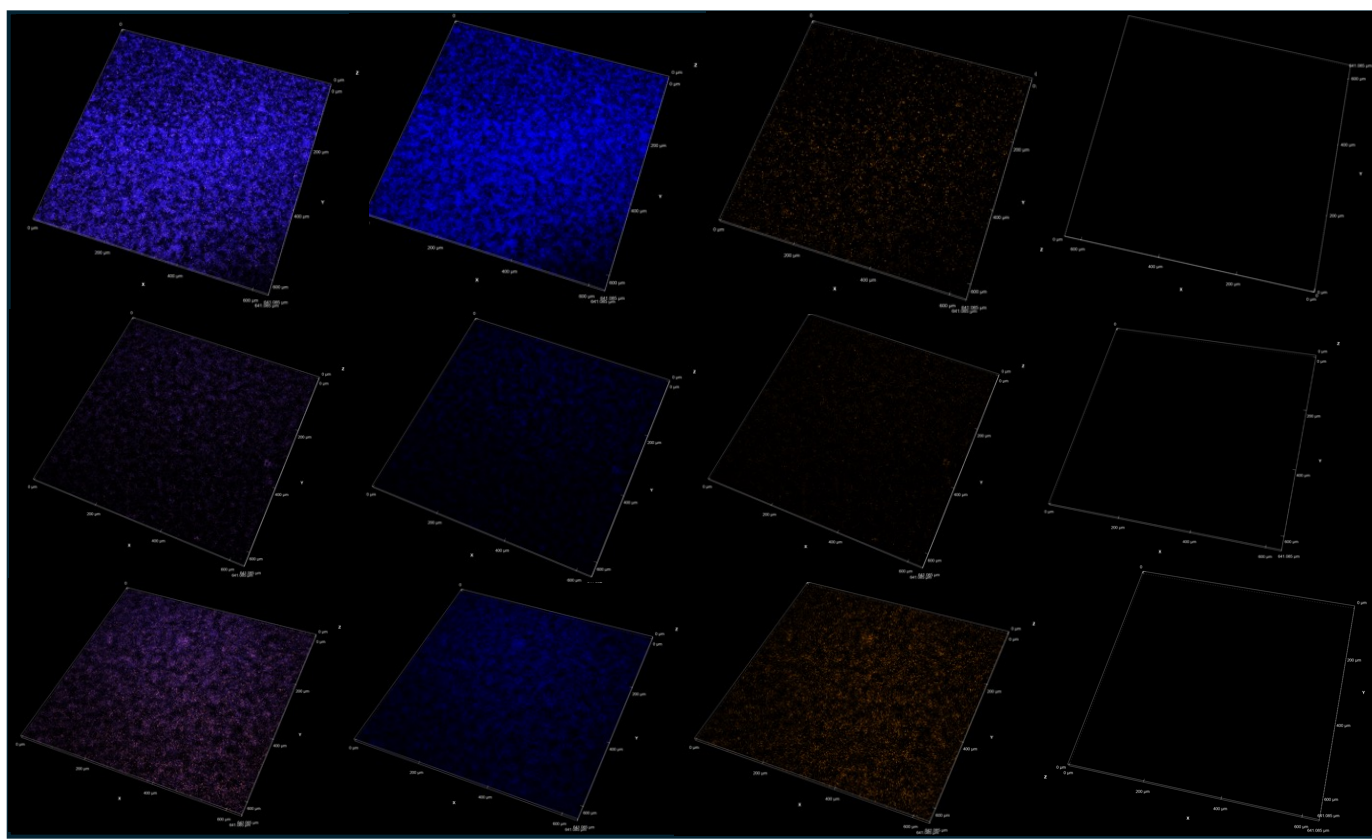

Supplement: Fig. S2 — PAO1 biofilms do not auto-fluoresce. [file jb.00507-25-s0002.pdf]

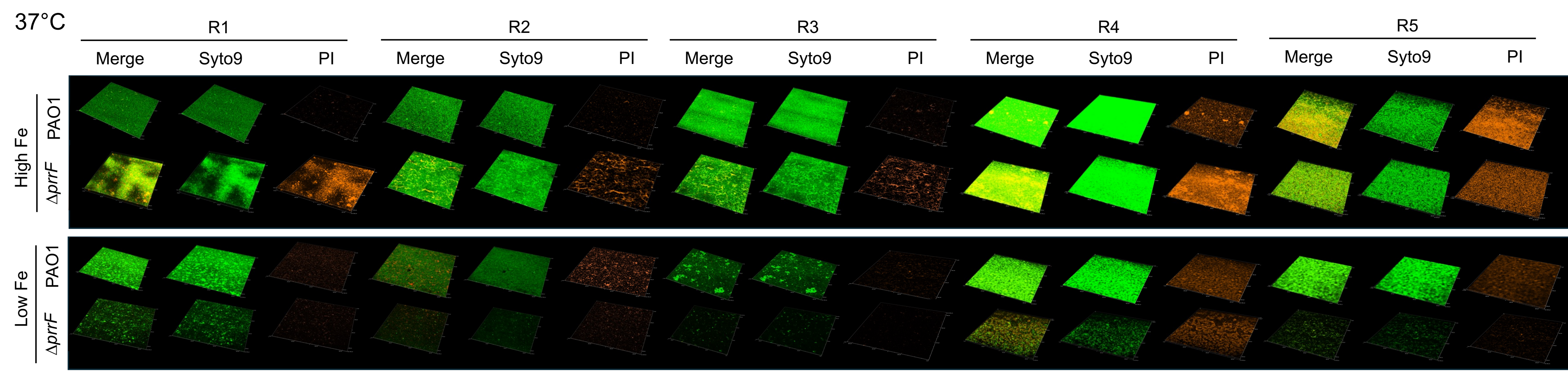

Supplement: Fig. S3 — Confocal images of all the biological replicates of biofilms of the indicated strains grown at 37°C. [file jb.00507-25-s0003.pdf]

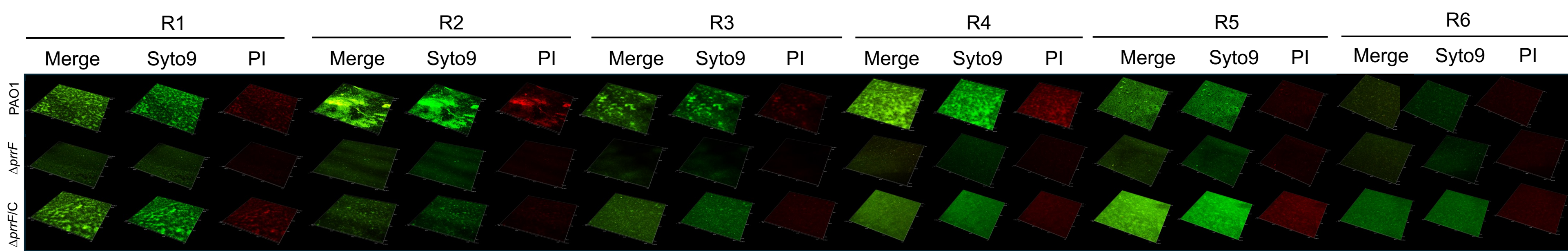

Supplement: Fig. S5 — Confocal images of biofilms of the indicated strains grown at 37°C. [file jb.00507-25-s0005.pdf]

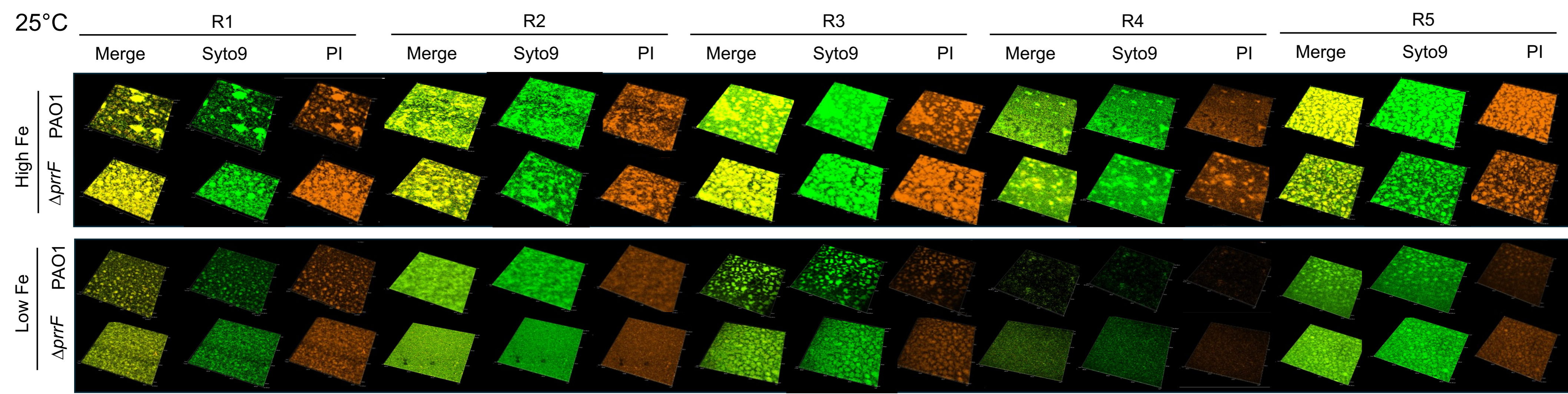

Supplement: Fig. S6 — Confocal images of biofilms of all the biological replicates of the indicated strains grown at 25°C. [file jb.00507-25-s0006.pdf]

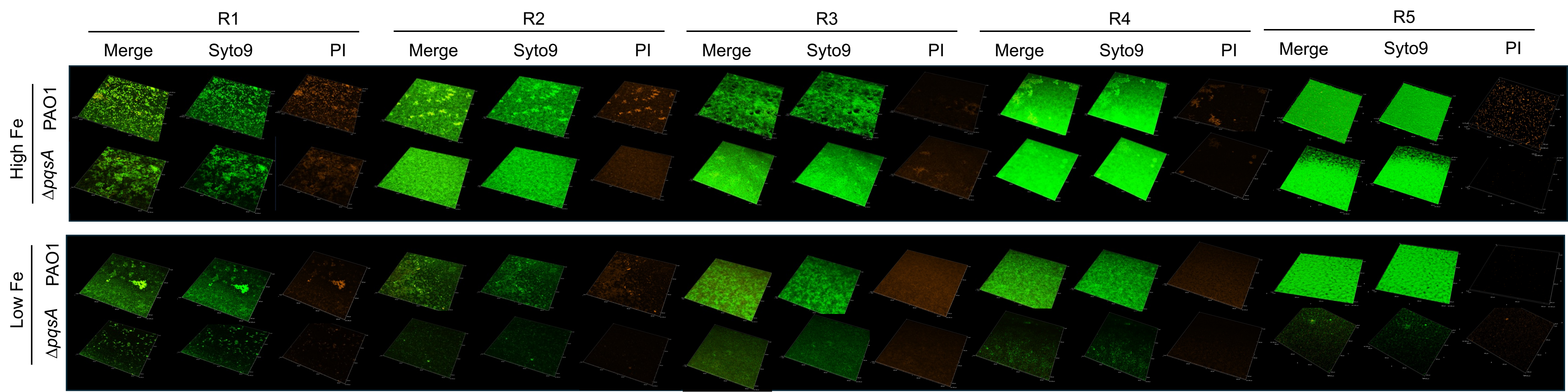

Supplement: Fig. S7 — Confocal images of biofilms of all the biological replicates of the indicated strains grown at 37°C. [file jb.00507-25-s0007.pdf]

High Fe

 $\Delta pq s A$ 

WT

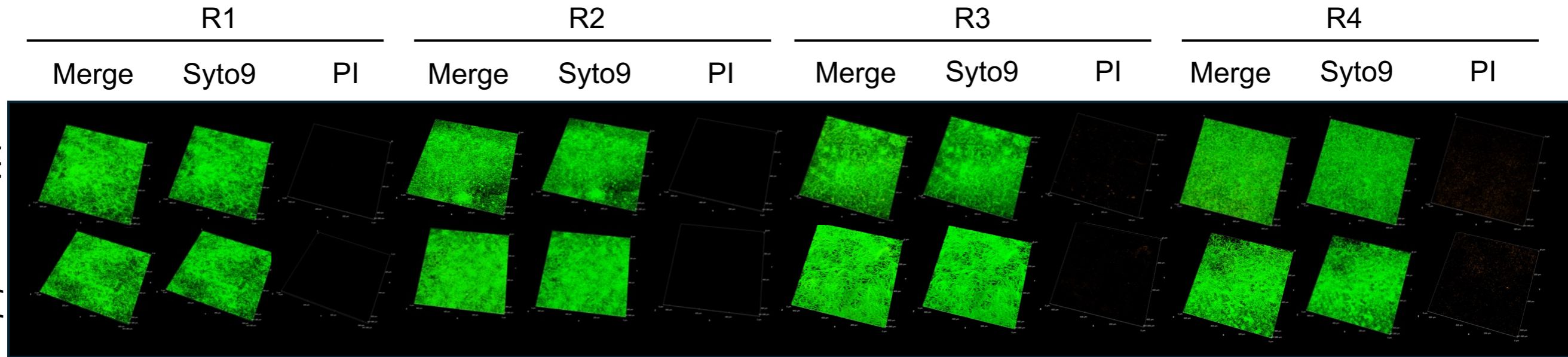

Low Fe

 $\Delta pq s A$ 

WT

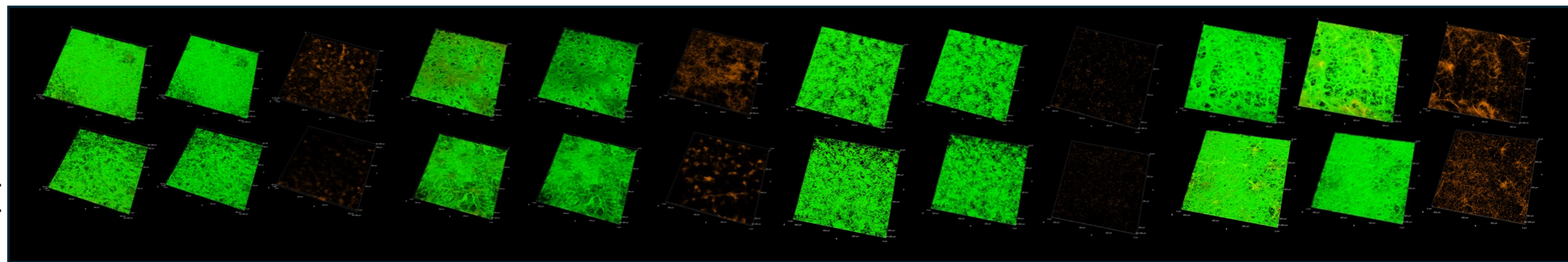

Supplement: Fig. S8 — Confocal images of biofilms of all the biological replicates of the indicated strains grown at 25°C. [file jb.00507-25-s0008.pdf]

## A Transcriptional

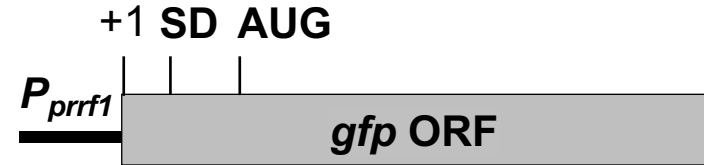

## B Transcriptional+Translational

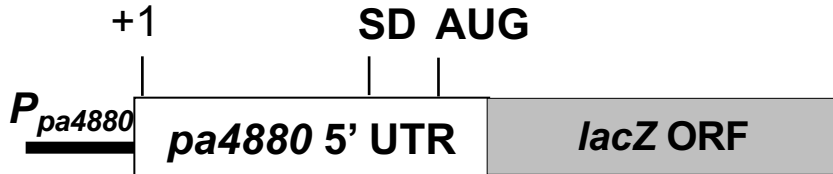

Supplement: Fig. S9 — Schematic of new reporter constructs. [file jb.00507-25-s0009.pdf]
