# Supplementary material for: The Pseudomonas aeruginosa PrrF sRNAs and PqsA promote biofilm formation at body temperature
Source: J Bacteriol. 2026 Jan 30;208(2):e00507-25. doi: 10.1128/jb.00507-25 (PMC12918728; doi:10.1128/jb.00507-25)
Supplement: Fig. S4 — Confocal images of all the biological replicates of time course biofilms of the indicated strains grown at 37°C. [file jb.00507-25-s0004.pdf]

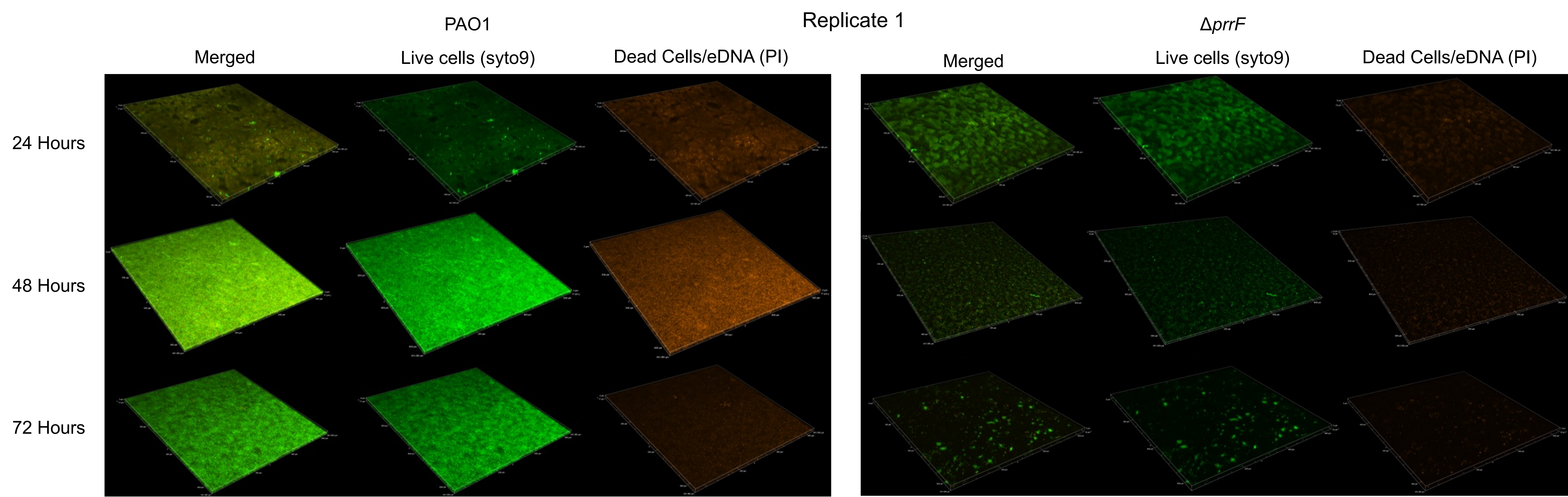
Replicate 1
PAO1
ΔprrF
Merged
Live cells (syto9)
Dead Cells/eDNA (PI)
Merged
Live cells (syto9)
Dead Cells/eDNA (PI)
24 Hours
48 Hours
72 Hours

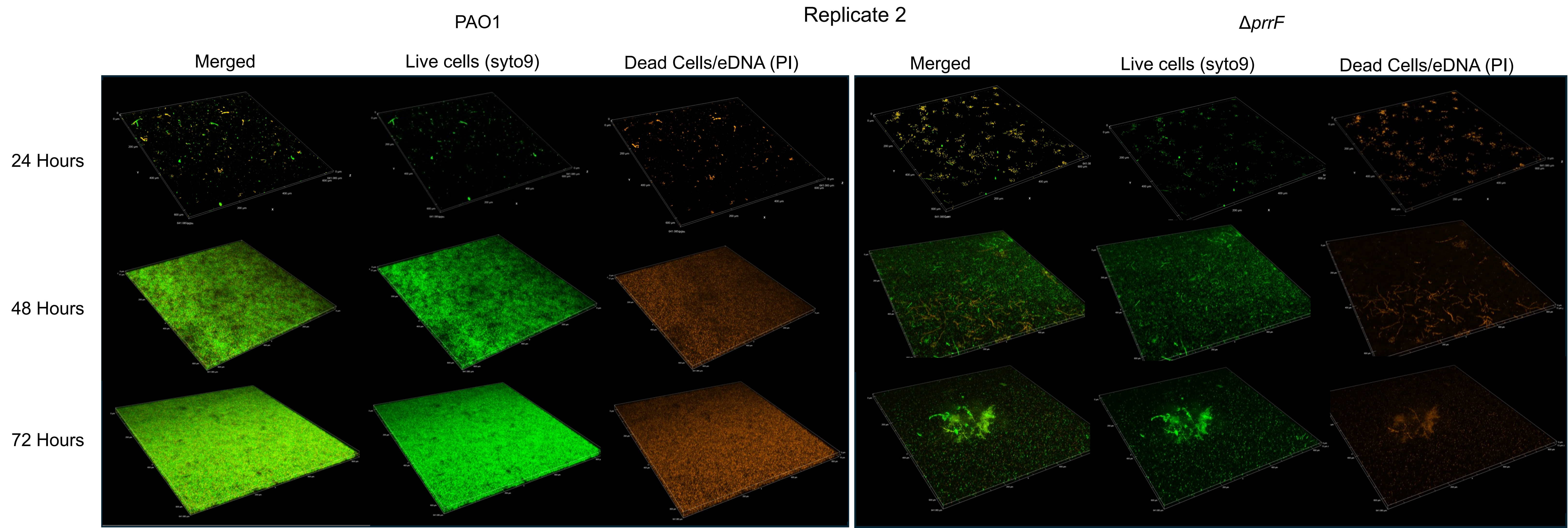
Replicate 2
PAO1
ΔprrF
Merged
Live cells (syto9)
Dead Cells/eDNA (PI)
Merged
Live cells (syto9)
Dead Cells/eDNA (PI)
24 Hours
48 Hours
72 Hours

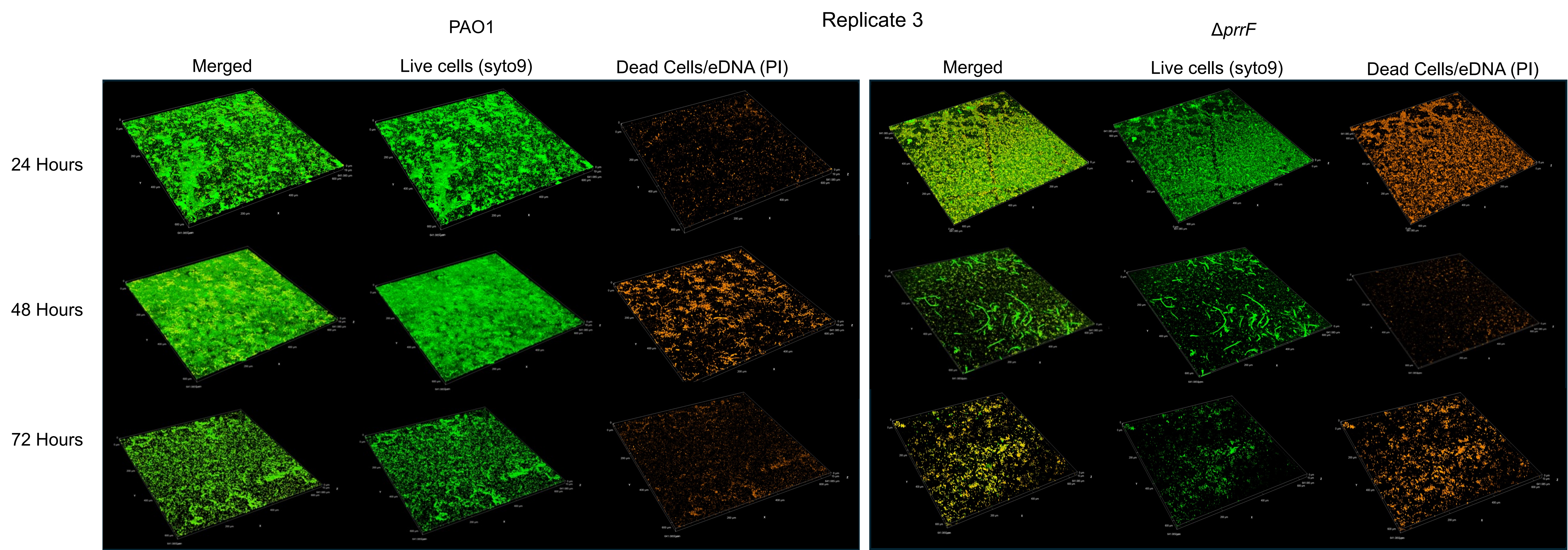

Replicate 3
PAO1
ΔprrF
Merged
Live cells (syto9)
Dead Cells/eDNA (PI)
Merged
Live cells (syto9)
Dead Cells/eDNA (PI)
24 Hours
48 Hours
72 Hours

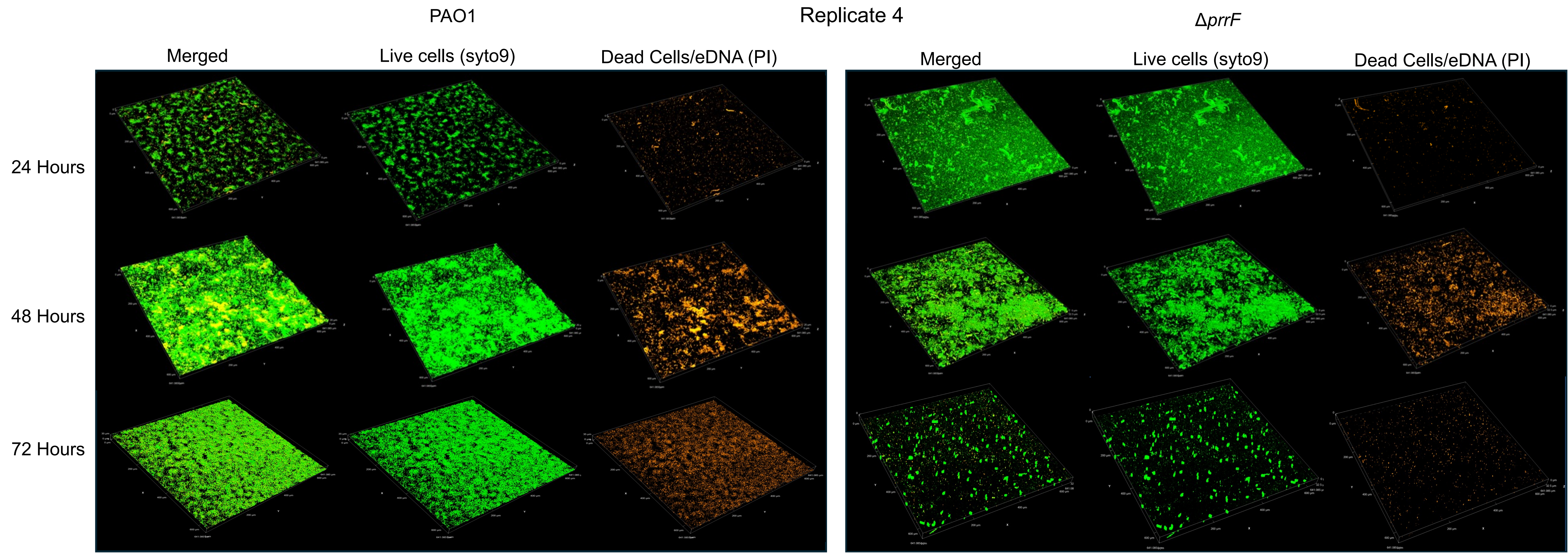
Replicate 4
PAO1
ΔprrF
Merged
Live cells (syto9)
Dead Cells/eDNA (PI)
Merged
Live cells (syto9)
Dead Cells/eDNA (PI)
24 Hours
48 Hours
72 Hours

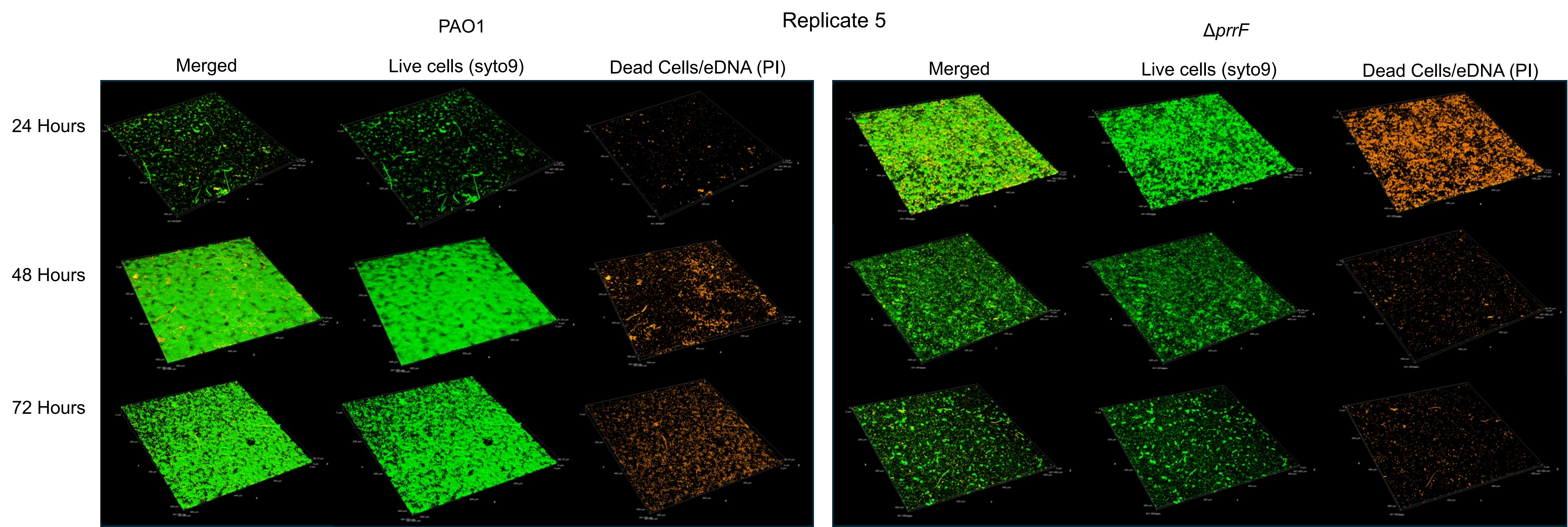
Replicate 5
PAO1
ΔprrF
Merged
Live cells (syto9)
Dead Cells/eDNA (PI)
Merged
Live cells (syto9)
Dead Cells/eDNA (PI)
24 Hours
48 Hours
72 Hours
