# Supplementary material for: The Pseudomonas aeruginosa PrrF sRNAs and PqsA promote biofilm formation at body temperature
Source: J Bacteriol. 2026 Jan 30;208(2):e00507-25. doi: 10.1128/jb.00507-25 (PMC12918728; doi:10.1128/jb.00507-25)
Supplement: Supplemental material — Legends for Figures S1 to S9, and Tables S1 and S2. [file jb.00507-25-s0010.pdf]

Supplementary materials for

The *Pseudomonas aeruginosa* PrrF sRNAs promote biofilm formation at body temperature

Rhishita Chourashi[1], Jacob M. Weiner[1], Tra-My Hoang[1&], Khady Ouattara[1], and Amanda G. Oglesby[1,2]

[&]Current address:

University of Maryland, Baltimore, [1]School of Pharmacy, [1]Department of Pharmaceutical Sciences and [2]School of Medicine, Department of Microbiology and Immunology, Baltimore, Maryland, 21201

Running title: The PrrF sRNAs contribute to biofilm formation at 37°C

Keywords: iron regulation, temperature regulation, PrrF, type IV pili, biofilm, *Pseudomonas aeruginosa*

*To whom correspondence should be addressed: aoglesby@rx.umaryland.edu

SUPPLEMENTARY FIGURE LEGENDS

Fig. S1. Confocal images showing iron dependent regulation of the *prrF1* promoter in flow-cell biofilms. Merged and individual channels of Syto9- and PI-stained biofilms of the P_{prrF1}-*gfp* reporter strain grown at 37°C used for quantification in **Fig. 1**. PI images are pseudo-colored orange.

Fig. S2. PAO1 biofilms do not auto-fluoresce. Confocal images of PAO1 biofilms grown at the indicated concentrations of iron at 37°C for 48h. Biofilms were stained with Hoechst 33342 (live cells) propidium iodide (PI, extracellular DNA). PI images are pseudo-colored orange.

Fig. S3. Confocal images of all the biological replicates of biofilms of the indicated strains grown at 37°C. Merged (A) and individual channels of Syto9- (B) and PI- (C) stained 37°C biofilms used for quantification in **Fig. 2B**. PI images are pseudo-colored orange.

Fig. S4. Confocal images of all the biological replicates of time course biofilms of the indicated strains grown at 37°C. Merged (A) and individual channels of Syto9- (B) and PI- (C) stained 37°C biofilms used for quantification in **Fig. 2D**. PI images are pseudo-colored orange.

Fig. S5. Confocal images of biofilms of the indicated strains grown at 37°C. Merged (A) and individual channels of Syto9- (B) and PI- (C) stained 25°C biofilms used for quantification in **Fig. 3**.

Fig. S6. Confocal images of biofilms of all the biological replicates of the indicated strains grown at 25°C. Merged (A) and individual channels of Syto9- (B) and PI- (C) stained 25°C biofilms used for quantification in **Fig. 2E**. PI images are pseudo-colored orange.

Fig. S7. Confocal images of biofilms of all the biological replicates of the indicated strains grown at 37°C. Merged (A) and individual channels of Syto9- (B) and PI- (C) stained 37°C biofilms used for quantification in **Fig. 4B**. PI images are pseudo-colored orange.

Fig. S8. Confocal images of biofilms of all the biological replicates of the indicated strains grown at 25°C. Merged (A) and individual channels of Syto9- (B) and PI- (C) stained 25°C biofilms used for quantification in **Fig. 4C**. PI images are pseudo-colored orange.

Fig. S9. Schematic of new reporter constructs. A. The *prrF1* promoter (P_{prrF1}) is fused with promoterless *gfp*. B. The *pa4880* promoter and 5' untranslated region is fused with *lacZ*. Reporter constructs were integrated into the *attB* sites of the PAO1 and ∆*prrF* chromosomes as described in the Materials and Methods.

SUPPLEMENTARY TABLES

Table S1. Strains and plasmids used in this study

Name	Description	Reference
Plasmids		
mini-CTX-*lacZ*	Integration-proficient plasmid containing a promoterless *lacZ* gene	Hoang, 2000 (1)
mini-CTX1-P_{pa4880}::*lacZ*$^{-SD}$	Mini-CTX1-*lacZ*$^{-SD}$ with the *pa4880* promoter (50 nt upstream of the transcriptional start site) + UTR cloned into the MCS	This study
mini-CTX-P_{prrF1}::*gfp*	Mini-CTX1-*gfp* with the *prrF1* promoter	This study
Strains		
DH5α	F– Φ80lacZΔM15 Δ(*lacZYA-argF*) U169 *recA1 endA1 hsdR17* (rK–, mK+) *phoA supE44* λ– *thi-1 gyrA96 relA1*	Taylor, 1989 (2)
SM10	*E. coli* used for conjugation: *pirR6K*	Taylor, 1989 (2)
SM10/pFLP	SM10 carrying the pFLP recombinase	Hoang, 1998 (3)
PAO1	*P. aeruginosa* laboratory strain	Holloway, 1955 (4)
PAO1/P_{prrF1}::*gfp*	PAO1 with the P_{prrF1}::*gfp* reporter integrated at the chromosomal *attB* site	This study
Δ*prrF*	*prrF1,2* deletion in PAO1	Wilderman, 2004 (5)
PAO1/vector	PAO1 carrying pUCP18	Reinhart, 2016 (6)
Δ*prrF*/vector	Δ*prrF1,2* mutant carrying pUCP18	Reinhart, 2016 (6)
Δ*prrF*/C	Δ*prrF1,2* mutant carrying pUCP18-*prrF1,2*	Reinhart, 2016 (6)
PAO1/P_{antR}::*lacZ*$^{-SD}$	PAO1 with the P_{antR}::*lacZ*$^{-SD}$ reporter integrated at the chromosomal *att* site	Djapgne, 2018 (7)
Δ*prrF*/P_{antR}::*lacZ*$^{-SD}$	Δ*prrF1,2* with the P_{antR}::*lacZ*$^{-SD}$ reporter integrated at the chromosomal *att* site	Djapgne, 2018 (7)
PAO1/P_{pa4880}::*lacZ*$^{-SD}$	PAO1 with the P_{pa4880}::*lacZ*$^{-SD}$ reporter integrated at the chromosomal *att* site	This study
Δ*prrF*/P_{pa4880}::*lacZ*$^{-SD}$	Δ*prrF1,2* with the P_{pa4880}::*lacZ*$^{-SD}$ reporter integrated at the chromosomal *att* site	This study
Δ*pilA*	*pilA* deletion in PAO1	C. Pritchett

Table S2. Primers used in this study

Name	Sequence
PA4880-50 post-transcriptional fusion	
For pa4880-50	GAATTCTCGGCGGCGGGTCGATAAAACTTTT
Rev pa4880-50	AAGCTTTTGAACGGTGGTCATCGGTTTTCTC
P_{prrF1}::gfp transcriptional fusion	
pPrrF1 F	ACTCGAGAGCTCTAGAAGCTTGCGCCTGCGCCGCAGCGCCTG
pPrrF1 R	ATGTTTTTCCTCCTTAAGCTTGAGTGACATAATAATGATTC
qPCR	
PrrF1.for	AACTGGTCGCGAGATCAGC
PrrF1.rev	CCGTGATTAGCCTGATGAGGAG
PrrF1.probe	CCCACGCAGTCGGACTCTTCAGATT

SUPPLEMENTARY REFERENCES

1. Hoang TT, Kutchma AJ, Becher A, Schweizer HP. 2000. Integration-proficient plasmids for *Pseudomonas aeruginosa*: site-specific integration and use for engineering of reporter and expression strains. Plasmid 43:59–72.
2. Taylor RK, Manoil C, Mekalanos JJ. 1989. Broad-host-range vectors for delivery of TnphoA: use in genetic analysis of secreted virulence determinants of Vibrio cholerae. J Bacteriol 171:1870–8.
3. Hoang TT, Karkhoff-Schweizer RR, Kutchma AJ, Schweizer HP. 1998. A broad-host-range Flp-FRT recombination system for site-specific excision of chromosomally-located DNA sequences: application for isolation of unmarked *Pseudomonas aeruginosa* mutants. Gene 212:77–86.
4. Holloway BW. 1955. Genetic recombination in *Pseudomonas aeruginosa*. J Gen Microbiol 13:572–581.
5. Wilderman PJ, Sowa NA, FitzGerald DJ, FitzGerald PC, Gottesman S, Ochsner UA, Vasil ML. 2004. Identification of tandem duplicate regulatory small RNAs in *Pseudomonas aeruginosa* involved in iron homeostasis. Proc Natl Acad Sci U S A 101:9792–7.
6. Reinhart AA, Nguyen AT, Brewer LK, Bevere J, Jones JW, Kane MA, Damron FH, Barbier M, Oglesby-Sherrouse AG. 2017. The *Pseudomonas aeruginosa* PrrF small RNAs regulate iron homeostasis during acute murine lung infection. Infect Immun 85.
7. Djapgne L, Panja S, Brewer L, Gans J, Kane MA, Woodson SA, Oglesby-Sherrouse AG. 2018. The *Pseudomonas aeruginosa* PrrF1 and PrrF2 small regulatory RNAs (sRNAs) promote 2-alkyl-4-quinolone production through redundant regulation of the *antR* mRNA. J Bacteriol doi:10.1128/JB.00704-17.
